# Resting blood pressure modulates chest pain intensity in patients with acute myocardial infarction

**DOI:** 10.1097/PR9.0000000000000714

**Published:** 2019-04-10

**Authors:** Michal Granot, Pnina Dagul, Doron Aronson

**Affiliations:** a The Laboratory of Clinical Neurophysiology, The Rappaport Faculty of Medicine, Technion-Israel Institute of Technology, Haifa, Israel; b Faculty of Social Welfare and Health Studies, University of Haifa, Haifa, Israel; c Department of Cardiology, Rambam Health Care Campus, Haifa, Israel; d The Rappaport Faculty of Medicine, Technion-Israel Institute of Technology, Haifa, Israel

**Keywords:** Pain modulation, Experimental pain, Blood pressure, Hypertension-induced hypoalgesia, Acute myocardial infarction

## Abstract

**Introduction and Objectives:**

Animal models and human studies show that resting blood pressure (BP) is inversely associated with pain sensitivity. The phenomenon of “hypertension-associated hypoalgesia” was proposed as a possible explanation for the intervariability in pain perception. Given that a portion of patients with acute myocardial infarction (AMI) do not experience significant pain, we used the model of severe cardiac ischemia to explore whether BP affects the intensity of chest pain.

**Methods:**

Patients with AMI admitted to the cardiac intensive care unit with coronary catheterization-proven completely occluded coronary artery were included (n = 67). Resting BP at admission and 5 days after AMI was obtained. Participants reported chest pain intensity and underwent psychophysical evaluation including pain ratings for pressure, heat, and pinprick stimuli as well as temporal summation and conditioned pain modulation paradigms.

**Results:**

Patients with lower systolic BP (≤120 mm Hg) vs higher (≥140 mm Hg) reported higher chest pain scores at symptom onset (82.3 vs 61.7, *P* = 0.048) and during peak AMI (82.8 vs 57.5, *P* = 0.019). Higher pain ratings in response to pinprick stimulus were associated with lower BP at admission (analysis of variance *P* = 0.036). Patients with hypertension demonstrated lower pain sensitivity in response to pressure stimulation (531.7 ± 158.9 kPa/s vs 429.1 ± 197.4). No significant associations were observed between BP and the other assessed psychophysical measures.

**Conclusion:**

Study findings reinforce the phenomenon of hypertension-associated hypoalgesia through characterization of the association between BP and clinical pain experiences at onset and during AMI in a model of acute clinical pain.

## Abstract 2

Autonomic factors such as blood pressure may partly determine individual's pain modulation capacity. Attenuated pain sensitivity might account for painless acute myocardial infarction and delay in seeking medical help.

## 1. Introduction

Evoked pain results in increased blood pressure (BP). Interestingly, accumulating evidence indicates that pain perception and its modulation are associated with an individual's BP at rest. The underlying mechanisms for this link, termed “hypertension-associated hypoalgesia,”^14^ are the activation of the autonomic nervous system and the inhibitory descending pain pathways, which couple higher arterial BP and heart rate with elevated pain threshold, and consequently produce a decreased pain experience.^4,5,14,23,32^

Animal studies demonstrated that acute and chronic hypertension (HTN) were associated with hypoalgesia.^12,14,30,31^ For example, several works^14,20,21,26,32^ compared normotensive and spontaneously hypertensive rats by exploring spinal nociceptive transmission in wide-dynamic-range neurons (sensitive to noxious and innocuous stimuli) and high-threshold neurons, suggesting that their responses were delayed and less intense in the hypertensive rats.

In humans, resting BP was inversely correlated with acute pain sensitivity in healthy normotensive subjects.^24^ Also, presurgical resting systolic and diastolic BP were negatively associated with acute postoperative pain intensity.^3,10,13^ Interestingly, the hypoalgesia–hypertension association has been predominantly observed in acute pain condition, and the link between BP and chronic pain remains to be determined.^8^ It has been suggested that hypertension altered the sensitivity of baroreceptors, which, in turn, impairs the nociceptive pathways of the pain regulatory processes,^4,8,9,23,25^ and particularly of the endogenous opioid system,^4,5^ potentially through activation of vagal afferents.^1,2,26,27^ Bruehl et al.^5,6^ supported the mediation effect of chronic pain on the presence of hypertension and suggested that it was due to reduced heart rate variability or baroreceptor activity associated with plastic alterations of pain inhibitory systems. Similar findings were observed in subjects with a history of chronic visceral pain during childhood, suggesting that plastic changes that occur in chronic pain conditions play a role in dysfunctional pain modulation.^6^

Given that pain modulation profile can be individually obtained using advanced psychophysical evaluation of pain pathways,^37^ it can be hypothesized that such evaluation approach can be used to better illuminate whether alteration of pain processing during activation of the nociceptive system, such as acute pain condition, is linked with resting BP. To address this question, we used the model of acute myocardial infarction (AMI) to explore hypertension-associated hypoalgesia.

Acute myocardial infarction represents a unique model of acute pain, although remarkably variable in its manifestation. Prolonged patient delay in seeking medical help during AMI emphasizes the severe consequences of attenuated pain sensation in such a critical situation because it may lead to a diminished perception of hazard and consequently delay effective treatments.

Previous work has focused on patients with diagnosis of silent myocardial infarction (SMI) to reveal whether higher resting BP affected their attenuated pain perception. Indeed, the diagnosis of SMI is nearly twice as common in hypertensive than in normotensive patients.^19^ In addition, attenuated pain response to tooth pulp testing characterized patients who had SMI.^11^ Likewise, among patients with stable angina, higher resting BP was linked with attenuation of pain intensity and unpleasantness scores in response to heat pain stimuli.^30^ We have reported that lower pain scores in response to contact heat stimuli and higher pain threshold characterize patients with painless AMI.^16.^ Nevertheless, it should be noted that such model of transient- or exercise-induced ischemia represents a different condition of chest pain, and the effect of BP on acute pain variability has not yet been explored in naturally occurring severe ischemic episode.

To further illuminate the phenomenon of “hypertension-associated hypoalgesia” in this particular setting, this study aimed to explore the role of resting BP on the intervariability in the perception of both clinical and experimental pain. To this end, we investigated the associations between pain modulation functioning as obtained by wide battery of psychophysical evaluation as well as chest pain scores at onset and during ST-elevation AMI.^17^

## 2. Patients and methods

### 2.1. Study population

Patients admitted to the cardiac intensive care unit of the Rambam Health Care Campus, Haifa, Israel, with the diagnosis of acute ST-elevation myocardial infarction (STEMI) enrolled in this study. The study sample consisted of the same patient cohort that were included in our previous study (for more details, see the study by Granot et al.,^17^ 2015). The diagnosis of STEMI was made based on the universal definition of myocardial infarction: (1) ischemic symptoms lasting for at least 20 minutes; (2) new or presumed new ST segment elevation at the J point in 2 or more contiguous leads with the cutoff points ≥0.2 mV in leads V1, V2, or V3, and ≥0.1 mV in other leads; and (3) typical rise of biochemical markers of myocardial necrosis.^34^ The investigational review committee on human research approved the study protocol (#3160), and each patient signed informed consent before the start of any experimental protocol.

Inclusion criteria were: (1) percutaneous coronary intervention within 24 hours from symptom onset, (2) presence of a completely occluded artery demonstrated during a coronary catheterization (Thrombolysis in Myocardial Infarction grade 0), which entailed the presence of an ischemic pain (not an independent inclusion criteria), and, (3) age above 30 and below 80 years. Exclusion criteria were: (1) previous myocardial infarction or stable angina; (2) use of analgesic or psychiatric medication on a regular basis (opioids, nonsteroidal anti-inflammatory drugs, selective serotonin noradrenalin reuptake inhibitors, pregabalin, and gabapentin); (3) inability to communicate and understand the instructions of the study; (4) hemodynamic instability; (5) chest pain during the 24 hours before the experimental pain tests; (6) pacemakers or implantable defibrillators; (7) existence of motor, cognitive, or psychiatric limitation/disability; and (8) chronic pain disorders or cancer.

### 2.2. Study design

Data about enrolled patients' BP as well as information regarding the use of antihypertensive pharmacological treatment on regular basis were collected from medical records accessed through the hospital's computerized database. The systolic and diastolic BP at admission to the emergency department were retrieved from the medical records of the enrolled patients. Blood pressure data at admission were categorized into 3 groups according to the systolic BP (SBP) as (1) ≤ 120 mm Hg; (2) 120 < BP < 140 mm Hg; and (3) ≥140 mm Hg.

Four to five days after patients were hospitalized at the intensive care unit, clinical characteristics of chest pain were obtained, wherein patients were asked to recall levels of pain intensity at onset of symptoms as well as mean and peak pain/highest pain using numerical pain scores (NPS) ranging from 0, denoting “no pain,” to 100, denoting “the worst pain imaginable.” At this session, the quantitative sensory testing (QST) was performed, and SBP and diastolic BP (DBP) were also measured. This was done while patients were considered cardiovascular stable but were still under clinical monitoring and pharmacological treatment, particularly antihypertensive drugs, according to their current medical conditions. As such, a portion was under the influence of direct and/or indirect effects on the autonomic and cardiovascular systems. The total duration of the experimental session was approximately 1.5 hours.

### 2.3. Psychophysical assessments

To attain a comprehensive understanding about the functioning of the nociceptive system, we delivered various types of stimulus modalities in both static and dynamic QST measures. All experiments were conducted in the same setting by a single experimenter (P.D.) at the Department of Cardiology in a designated, comfortable, quiet room. To minimize the effect of analgesics on the accuracy of the psychophysical evaluation and to rule out the possible influence of current pain on the nociceptive functioning, patients were self-reported to be pain-free at the time of QST evaluation and were asked to refrain from pain-relief medications in the 2 to 4 hours preceding the experimental pain trial.

#### 2.3.1. Pressure pain threshold

Pressure stimulus of increasing intensity was applied to the volar aspect of the right forearm using a pressure algometer (Somedic, Horby, Sweden) with a probe diameter of 1 cm. Patients were instructed to press the “stop” button when the stimulus was first perceived as painful. Pressure pain threshold (PPT) was calculated by averaging the pain threshold pressure (kilopascal) of 4 successive trials.

#### 2.3.2. Mechanical pinprick pain score and mechanical temporal summation

To evoke mechanical temporal summation, a train of 10 identical pinprick stimuli was delivered to the right volar forearm using von Frey monofilaments of 6.45 nM (225.1 g). The stimuli were administered within an area of 1 cm^2^. Each patient was exposed to a single stimulus and then asked to rate the level of pinprick pain intensity using the NPS. Subsequently, 10 repetitive stimuli with an interstimulus interval of 1 second were applied within an area of 1 cm in diameter using the same filament. Patients were then asked to rate the pain intensity of the last stimulus. Pain scores in response to the first stimulus served as a measure for mechanical suprathreshold pinprick pain. The mechanical temporal summation value was calculated as the difference between the pain scores obtained for the last and first pinprick stimuli.

#### 2.3.3. Conditioned pain modulation evaluation

Conditioned pain modulation (CPM) assessment was performed using the parallel paradigm in which 2 identical “test-pain” stimuli were delivered before and simultaneous to noxious “conditioning stimulus.” The “test-pain” was a pressure stimulus applied to the volar aspect of the dominant hand for 30 seconds. The baseline was increased from 0 at a rate of 30 kPa/s. The “test-pain” was administered at the pain-60 intensity, which is the kilopascal value that induced a pain score of 60 on a 0 to 100 NPS. The level of pressure that was required to evoke pain-60 was considered to be an additional psychophysical measure indicating pain sensitivity. After a 15-second break, a contact heat stimulation of 46.0°C was delivered to the nondominant forearm for 60 seconds. During the first 30 seconds of the “conditioning stimulus,” the patients rated their pain intensity every 10 seconds, and the mean of pain ratings was taken as measure of heat pain perception. During the second 30 seconds of the “conditioning stimulus,” the “test-pain” was repeated; patients were then asked to rate the “test-pain” every 10 seconds as well. The CPM response was calculated as the difference between the “test-pain” and the “conditioned pain” for the average pressure pain scores before and during the “conditioning stimulus.” The higher the negative value, the more efficient the CPM is.

### 2.4. Statistical analyses

All statistical analyses were performed using SPSS (version 23; SPSS Inc, Chicago, IL). Continuous variables are presented as mean (±SD) and categorical variables as frequency numbers (percentages). Scaled variables were tested for normality using the Kolmogorov–Smirnov test. To investigate the associations between BP and pain parameters, for normally distributed variables, comparisons of means between the aforementioned 3 BP groups were performed using analysis of variance (ANOVA) with a linearity test and Tukey post hoc analyses. For nonnormally distributed variables, the Kruskal–Wallis ANOVA was used to compare the 3 groups. Spearman rank–order correlations were calculated between the clinical pain measures (pain at onset and during peak MI) and the experimental pain measures and SBP or DBP.

## 3. Results

### 3.1. Description of the study population

**Figure 1.**
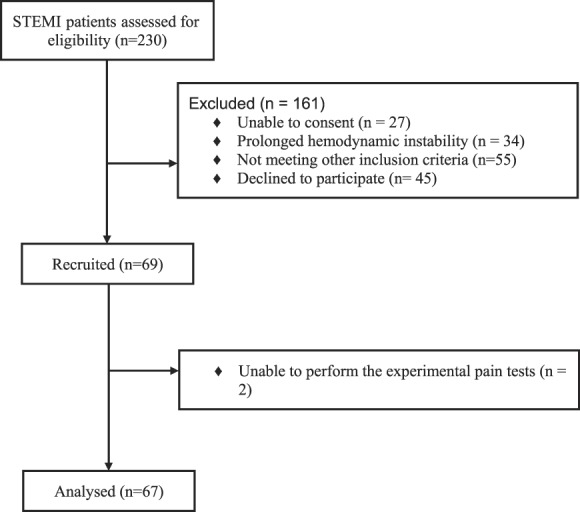
Flow diagram of patient enrollment.

During the study period, 230 patients with STEMI were hospitalized in the intensive care unit of the Rambam Health Care Campus. Of those, 163 patients were excluded for various reasons (Fig. 1). Therefore, the final study cohort consisted of 67 patients (for more details about the cohort, see the study by Granot et al.,^17^ 2015). The participants were divided into 3 subgroups according to their BP at admission to the hospital. This number is considered clean of any medication influence that can affect the cardiovascular system. Systolic BP categories included (1) patients with levels ≤120 mm Hg, (2) patients with 120 <SBP <140, and (3) patients with ≥140 mm Hg. In addition, the sample was divided into 2 groups according to their diagnosis of hypertension (53%) or normotensive (47%). The clinical and demographic characteristics of the study population are presented in Table 1.

**Table 1:**
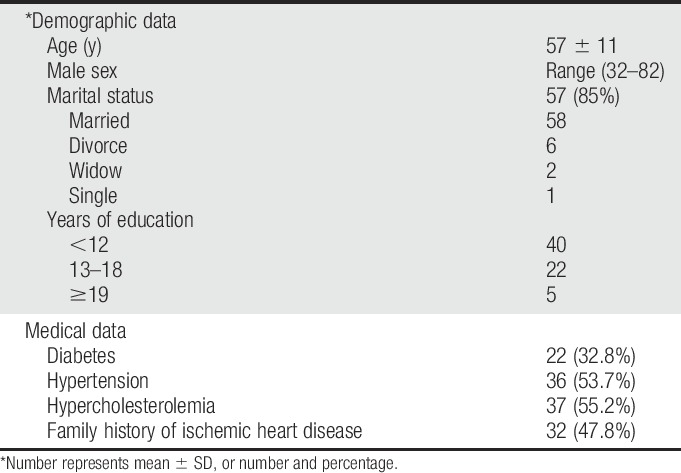
Demographic and clinical data.

Analysis of variance revealed no differences (*P* = 0.724) in SBP and DBP values measured after hospitalization between the 3 SBP categories. This may indicate that these 2 assessments of BP as obtained at admission or after 5 days of hospitalization were not correlated, and eventually represent 2 different situations. In the same line, a prior diagnosis of HTN was not associated with the SBP category on admission (χ^2^ = 4.366; *P* = 0.113).

### 3.2. Clinical chest pain characteristics

The mean pain intensity at symptom onset was 62.2 ± 26.3 NPS. Mean pain scores were 75.4 ± 20.8, and pain intensity ratings at peak were 78.6 ± 21.7. Pain scores at symptom onset were correlated with peak pain scores (*r* = 0.993; *P* < 0.001). Patients described the dominant quality of their chest pain as pressure (61.5%), burning (23.1%), stabbing (9.2%), and undefined (6.2%). Most of the patients (65.7%) reported that the pain was radiating, predominantly to the left arm, back, or lower jaw.

### 3.3. Association between systolic blood pressure and clinical chest pain

**Figure 2.**
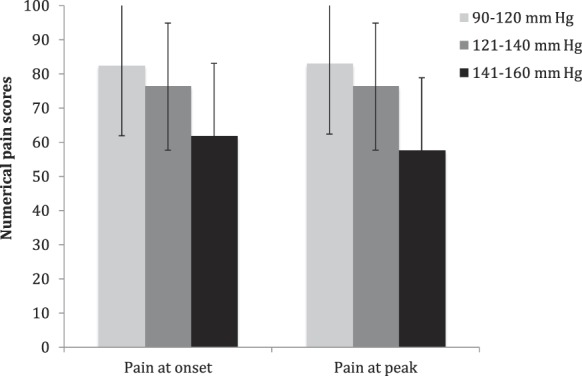
Clinical chest pain characteristics (at symptom onset and at peak pain) categorized according to BP levels. BP, blood pressure.

The ANOVA model demonstrated a significant linear effect of BP on perceived chest pain intensity both at symptom onset (*P* = 0.019) and peak pain intensity (*P* = 0.007) (Fig. 2). However, no significant correlation was found between SBP obtained during hospitalization and the intensity of chest pain at symptom onset (*r* = 0.089; *P* = 0.477), nor for the pain peak intensity (*r* = 0.003; *P* = 0.984). Likewise, no correlations were observed between SBP or DBP values and any of the psychophysical pain tests, nor with the clinical chest pain reports at any point. Interestingly, the incidence of patients who reported radiating pain (the left hand) was greater among the patients with no diagnosis of hypertension compared with hypertensive patients (χ^2^ = 3.88; *P* = 0.049).

### 3.4. Association between blood pressure and experimental pain

Higher chest pain ratings obtained at onset of symptoms as well as at peak were correlated with higher intensities of pressure required to evoke pain that was perceived as 60 on the 0 to 100 NPS (*r* = 0.276, *P* = 0.035; *r* = 290, *P* = 0.026, respectively). The magnitude of CPM, TS, as well as pain scores in response to the heat or mechanical pinprick stimulation was not correlated with BP. In addition, no significant differences were found between the 3 SBP categories in relation to any of the other psychophysical measures.

Significant linear changes were found between the 3 BP groups for mechanical stimulation and PPT. Higher pinprick pain ratings were found among patients with lower BP at admission. Patients with SBP between 90 to 120 mm Hg rated this stimulus as 8.1 ± 11, whereas those with SBP between 120 to 140 mm Hg 3.5 ± 2.1 and patients with SBP above 140 mm Hg scored this stimulus as zero (*P* = 0.036).

A comparison between patients with and without diagnosis of hypertension revealed that lower intensities of pressure stimulation were required to evoke pain-60 experiences in the former as compared to the latter (429.1 ± 197.4 vs 531.7 ± 158.9 kPa/s, *P* = 0.036). However, no significant differences were observed in the clinical chest pain measures between patients with and without a medical diagnosis of hypertension.

## 4. Discussion

The model used in the current study to investigate hypertension-induced hypoalgesia is unique because it is the first to focus on AMI patients with a proven occluded coronary artery, which provokes severe hypoxia and ischemia, resulting in acute chest pain. This model allowed us to address the variability in pain perception by using a matrix that comprised BP measurements as well as a comprehensive psychophysical assessment. The main findings support previous reports that lower BP may affect pain modulatory processes of nociceptive input, resulting in hypoalgesia. Notably, as previously reported,^17^ no association was found between the intensity of chest pain and the severity of ischemic area, suggesting that those patients with lower BP did not report higher chest pain due to a more massive cardiac ischemia.

The domain of pain variability in the context of cardiac ischemia has been studied for more than 3 decades, starting with the hypertensive animal models.^22,32^ Later on, Zamir and Maixner^38^ suggested that altered regulatory processing of the cardiovascular system in response to stressors affects pain perception. Sheps et al.^32^ proposed in an editorial several possible mechanisms that affect chest pain variability in myocardial ischemia. For example, stimulation of the volume heart baroreceptors may trigger vagal activation and circulating opioids.

Although previous studies^5,24,25,34,36,38^ established that BP affects pain, the models used mainly experimental measures of pain thresholds obtained in healthy subjects by experimentally induced pain or in SMI patients in whom pain was induced by a treadmill exercise. Thus, the concept that baroreflex sensitivity is linked with elevated resting BP and both are involved in hypoalgesia through diminished central sensitization and enhanced descending inhibition necessitates further illumination.

In terms of pain responses preceding BP patterns, France et al.^12^ reported that hypoalgesia anteceded hypertension in normotensive persons with a family history of hypertension, and Campbell et al.^7^ concluded that pain tolerance measured at 14 years of age predicts ambulatory BP at the age of 22 years. The association between pain perception and BP was delineated in a clinical cohort of chronic pain by Granot et al.,^14,15^ in which vulvar pain characteristics were affected by higher systolic and diastolic BP.

The observation in animals that hypoalgesia is present in young normotensive rats that later develop HTN^12,14^ emphasizes the link between the autonomic and nociceptive system. This finding calls for additional studies because it is not yet clear whether exaggerated hypoalgesia may serve as a predictive, pathophysiological marker that is associated with an increased risk of developing high BP, or vice versa.

Interestingly, BP values measured after hospitalization did not necessarily correspond with those obtained at admission. Furthermore, a prior HTN diagnosis was not associated with BP assessed at admission and after hospitalization. These gaps probably arise from the fact that posthospitalization BP was “corrected” with antihypertensive medications and was therefore noncomparable with all initial “natural” BP values. However, BP values with which patients were admitted to the hospital does reflect their pharmacologically less-affected hemodynamic state that reliably represents BP during the experienced chest pain at onset and at peak. This may explain why (1) only BP values on admission were associated with the patients' clinical pain, and (2) the absence of a correlation between the experimental pain measures and the clinical pain outcomes^17^ because they were collected several days after the MI had occurred. In this regard, reports on patients undergoing thoracotomy^35^ or suffering from diabetic neuropathy^36^ have also failed to establish an association between psychophysical measures and the perception of acute pain that is probably mediated by other mechanisms such as autonomic, metabolic, as well as cognitive factors. Nevertheless, the fact that those patients who necessitated higher pressure intensities to evoke pain-60 perception were of lower BPs might support the assertion that modulatory pathways may account for the variability of the perceived pain in the setting of AMI.

The current research may contribute to this line of research by focusing on a clinical acute pain condition, in which pain sensation is required to encourage defensive protective behavior, ie, seeking promptly medical assistance. In addition, this study in which dynamic pain measures that reflect ascending and descending pain pathways were evaluated using various pain modalities may open a wider window into the nociceptive processes that are assumed to be involved in the central mechanisms of pain.

### 4.1. Study limitations

Our study has several limitations. First, the relatively small sample size limits the generalization of our findings, given the variability in psychophysical assessment. This confinement was due to the restricted inclusion criteria, which decreased the number of patients that could be enrolled. Second, it is important to acknowledge the possibility that ongoing cardiac ischemia evoked nociceptive processes that affected the modulation system such that this process was also involved in the perception of pain—as an uncontrolled conditioning stimulation. Because the experimental pain was assessed a few days after hospital admission and all patients were pain-free during the tests, we assume that such processes may be relevant to the clinical pain reports, but not to the QST measures. Third, the test stimulus and the conditioning stimulus were delivered to both upper limbs. Thus, it may be possible that the CPM paradigm cannot be considered heterotopic because spinal segmental modulatory systems may bias the CPM response. However, a similar CPM paradigm in which the test stimulus and the conditioning stimulus were both delivered to upper limbs has been widely reported.^18^ Fourth, although a statistically significant correlation was found between higher chest pain ratings at onset and temperature required to evoke a pain-60 response, the association was weak and should be noted as such. Finally, patients were asked to recall the intensity of pain at symptom onset several days after the event, potentially introducing recall bias.

In conclusion, the current findings shed important light on the phenomenon of hypertension-associated hypoalgesia through characterization of the association between BP and clinical pain experiences at onset and during AMI in a model of acute clinical, yet spontaneous, pain. Accordingly, autonomic factors, and mainly BP, may partly determine an individual's pain modulation capacity, which dictates the large variability in pain symptoms.

## Disclosures

The authors have no conflict of interest to declare.
